# Tailored Hydrogels as Delivery Platforms for Conditioned Medium from Mesenchymal Stem Cells in a Model of Acute Colitis in Mice

**DOI:** 10.3390/pharmaceutics13081127

**Published:** 2021-07-23

**Authors:** Juan Sendon-Lago, Lorena Garcia-del Rio, Noemi Eiro, Patricia Diaz-Rodriguez, Leandro Avila, Luis O. Gonzalez, Francisco J. Vizoso, Roman Perez-Fernandez, Mariana Landin

**Affiliations:** 1 Center for Research in Molecular Medicine and Chronic Diseases (CIMUS), Department of Physiology, Universidade de Santiago de Compostela, Avda. de Barcelona 22, 15706 Santiago de Compostela, Spain; bautistax@hotmail.com (J.S.-L.); leandro.avila@usc.es (L.A.); 2 Department of Pharmacology, Pharmacy and Pharmaceutical Technology, Universidade de Santiago de Compostela, 15782 Santiago de Compostela, Spain; lorena.garcia.delrio@rai.usc.es (L.G.-d.R.); patricia.diaz.rodriguez@usc.es (P.D.-R.); 3 Research Unit, Hospital Fundación de Jove, Avda. Eduardo de Castro 161, 33290 Gijón, Spain; noemi.eiro@gmail.com (N.E.); investigacion@hospitaldejove.com (L.O.G.)

**Keywords:** experimental colitis, human uterine cervical stem cells, mucoadhesive and thermosensitive hydrogel, inflammation, conditioned medium delivery

## Abstract

Inflammatory bowel disease (IBD), including Crohn’s disease (CD) and ulcerative colitis (UC), is increasingly prevalent and current therapies are not completely effective. Mesenchymal stem cells are emerging as a promising therapeutic option. Here, the effect of local hydrogel application loaded with conditioned medium (CM) from human uterine cervical stem cells (hUCESC-CM) in an experimental acute colitis mice model has been evaluated. Colitis induction was carried out in C57BL/6 mice by dissolving dextran sulfate sodium (DSS) in drinking water for nine days. Ulcers were treated by rectal administration of either mesalazine (as positive control) or a mucoadhesive and thermosensitive hydrogel loaded with hUCESC-CM (H-hUCESC-CM). Body weight changes, colon length, and histopathological analysis were evaluated. In addition, pro-inflammatory TNF-α, IL-6, and IFN-γ mRNA levels were measured by qPCR. Treatment with H-hUCESC-CM inhibited body weight loss and colon shortening and induced a significant decrease in colon mucosa degeneration, as well as TNF-α, IFN-γ, and IL-6 mRNA levels. Results indicate that H-hUCESC-CM effectively alleviated DSS-induced colitis in mice, suggesting that H-hUCESC-CM may represent an attractive cell-free therapy for local treatment of IBD.

## 1. Introduction

Inflammatory bowel disease (IBD) represents a group of pathogenic, chronic, and degenerative disorders defined by recurrent inflammation of the gastrointestinal tract. The two major forms are ulcerative colitis (UC) and Crohn’s disease (CD). It is estimated that as many as 1.4 million Americans and 2.4 million Europeans are suffering from this disease [1]. In addition, the incidence and prevalence of IBD have increased dramatically over the last few decades, growing as a public health concern since they can lead to life-threatening problems [2,3]. IBD is multifactorial and its aetiology is not precisely known. IBD involves an abnormal systemic and mucosal immune response against intraluminal antigens, favoured by microbial factors and alterations of the mucosal barrier, in genetically predisposed individuals [4,5].

The current treatment for IBD begins with 5-aminosalicylic acids (5-ASA) and antibiotics. Mesalazine or 5-ASA is the first-line treatment for patients with IBD. It is administered orally (tablets or sachets) and/or topically (suppositories) in cases of mild to moderate active ulcerative colitis, depending on the severity and affected areas [6,7]. In patients with CD, mesalazine is administered orally to induce remission in patients with mild or moderate disease. Its use is controversial because some studies have shown that its therapeutic effect is not clinically relevant [8]. Patients who do not respond to 5-ASA are treated with corticosteroids, immunomodulators, and biologic agents. When all else fails, patients undergo surgery [9]. However, current therapies are often not completely effective and may have side effects related to the gastrointestinal tract and liver. Therefore, there is great interest in the development of more effective drugs for IBD treatment [10].

Mesenchymal stem cells (MSCs) are fibroblast-like and multipotent progenitors that can be isolated from the connective tissue of most organs [11]. MSCs express defined surface markers, including CD90, CD73, and CD105, and present immuno-regulatory and regenerative properties. MSCs are considered a promising medical alternative for the treatment of various inflammatory and autoimmune diseases. They have been shown to target injured tissue, secrete paracrine anti-inflammatory factors, and induce inhibition of T-cell proliferation, B-cell function, and dendritic cell maturation, via secretion of soluble factors [12]. Therefore, stem cell therapies have emerged as novel therapeutic tools for IBD patients who respond poorly to conventional anti-inflammatory treatments [13]. Several studies have demonstrated that MSCs from different origins ameliorate certain parameters such as body weight, bleeding, stool consistence, mortality rate, colon length, inflammation-associated histological changes, and inflammatory cytokine expression in experimental colitis [14]. Furthermore, products derived from MSCs such as conditioned medium (CM), MSC extracts, and exosomes have also been tested [15,16,17]. Clinical trials suggest that local application of MSCs is a safe and beneficial therapeutic approach for the healing of perianal fistulas in CD patients [18,19]. Therefore, MSCs may represent a promising therapy for IBD. Nevertheless, many questions concerning the optimal origin and source of MSCs, dosage, and routes of administration remain unresolved.

A new population of MSCs, called human uterine cervical stem cells (hUCESCs), which are obtained from the transition zone of the uterine cervix of healthy women through Pap cervical smears, has been isolated and characterized [20]. hUCESCs present a high rate of proliferation in culture, with a doubling index of 1.76 in 24 h. Conditioned medium from hUCESCs (hUCESC-CM) displayed a potent antitumor effect against breast and cervical cancer cells, and also against stromal cells by inhibiting the proliferation and invasion of cancer-associated fibroblasts (CAFs), and inhibiting and reverting monocyte to macrophage differentiation [20]. Recent studies carried out previously have also demonstrated the beneficial effects of hUCESC-CM on corneal wound healing and ocular inflammation in rat and rabbit models, as well as its anti-microbial properties [21,22,23,24]. The anti-inflammatory and regenerative properties of hUCESC-CM could be of particular interest for the management of IBD, if properly applied on the colonic mucosa.

Hydrogels are 3D colloidal network structures widely used in the pharmaceutical field due to their similitude to extracellular matrices and biocompatibility. Their aqueous environment allows oxygen permeability and the diffusion of nutrients and small molecules [25,26]. Their properties suggest that they can be excellent carriers to evaluate the potential usefulness of hUCESC-CM to restore damaged rectal and colonic mucosae in IBD. Hydrogels are capable of spreading over ulcerated mucous membranes and release the hUCESC-CM active components to the epithelium while protecting mucosal surfaces from abrasion, which should favor their rapid regeneration. To this end, the design of thermosensitive and mucoadhesive hydrogels as platforms to deliver hUCESC-CM to the colonic area is proposed. Based on the authors previous work [27], two Pluronic^®^ and a hydroxypropylcellulose (MK4M) have been selected. Pluronics^®^ are amphiphilic, thermosensitive, and bioadhesive triblock copolymers widely used to produce in situ gelation hydrogels [27]. Often, PF127 and PF68 varieties are used in combination to obtain hydrogels with specific gelation temperatures [28]. Hydroxypropylcellulose is a cellulose derivative with a high viscosity grade that generates robust and mucoadhesive networks capable of maintaining hydrogel integrity or even controlling the release of drugs. Thus, the combination of these compounds should increase the binding of the hydrogel to the colonic and rectal mucosae and its protective capacity [29].

In the present study, involving a DSS-induced colitis mice model, the effectiveness of an optimized thermosensitive and highly mucoadhesive hydrogel, loaded with hUCESC-CM, was evaluated as a topical delivery platform to increase hUCESC-CM residence time and morphologically repair the damaged tissue in the colonic area.

## 2. Materials and Methods

### 2.1. Materials

Pluronic^®^ F127 (PF127) and F68 (PF68), Fetal Bovine Serum (FBS), Dulbecco’s Modified Eagle Medium: Nutrient Mixture F-12 (DMEM-F12), and phosphate buffered saline (PBS) were acquired from Sigma-Aldrich (St. Louis, MO, USA). Methocel^®^ K4M (MK4M) was purchased from Dow (Midland, MI, USA). Round tip 24 G × 1.5″ needles were obtained from PetSurgical (Phoenix, AZ, USA). Dextran sulfate sodium (DSS) was acquired from MP Biomedicals (Illkirch, France). Mesalazine (Claversal foam, 1 g) was provided by FAES FARMA (Madrid, Spain).

### 2.2. Hydrogel Development and Preparation

A mucoadhesive and thermosensitive hydrogel suitable for rectal administration was developed and optimized using INForm^®^ v.5.01 software (Intelligensys Ltd., Stokesley, UK) that combines Artificial Neural Networks (ANN) and genetic algorithms (GA). PF127, PF68, and MK4M were selected as formulation components based on their thermosensitive and bioadhesive properties [22]. A Balance Density Experimental Design (DataForm^®^ v3.1, Intelligensys Ltd., Stokesley, UK) was established to analyze the influence of each ingredient on several parameters (syringeability, bioadhesion, and gelation temperature (Tgel). After ANN modelization, the model was asked for a composition of a hydrogel that simultaneously had a syringeability lower than 86 mJ, the highest bioadhesion over 0.254 mJ, and a Tgel in the range of 25–27 °C. In this way, the formulation would be easy to apply rectally through the catheter (24 G × 1.5″) with a very small internal diameter. It would spread widely on the mucosa and would gel quickly to avoid losses and favor the superficial protection of the tissue. In addition, its mucoadhesion characteristics would facilitate its permanence in the action site. The composition selected by Genetic Algorithms as optimal hydrogel formulation by the ANN model and GA includes 16.34% of PF127, 2.55% of PF68, and 0.26% of MK4M.

The optimized hydrogel was prepared by dissolving accurate amounts of PF127 and PF68 in cold dissolution media at 4 °C overnight. Then, MK4M (%*w/w*) was added to this solution after prior cooling in an ice bath and dissolved under magnetic stirring until a homogeneous and clear solution was obtained. Hydrogel sterilization was carried out by autoclaving (Trade Raypa^®^ steam sterilizer AES-12, Barcelona, Spain). Sterile hydrogels were stored at 4 °C until use. Three dissolution media were selected to perform the current study: double distilled water (ddH_2_O), cell culture media (DMEM-F12), and phosphate buffered saline (PBS).

Hydrogels prepared with PBS (H-PBS) were used to validate the ANN model. Hydrogels prepared in cell culture media (H-DMEM-F12) were used as controls. Lyophilized hUCESC-CM was resuspended in the optimized hydrogel prepared with ddH_2_O to obtain the hUCESC-CM loaded hydrogel (H-hUCESC-CM).

### 2.3. Hydrogel Characterization

#### 2.3.1. Texturometric Analysis

Syringeability and bioadhesion were evaluated using a TA XT Plus Texture Analyser (Surrey, UK), following a protocol which was previously reported [27]. Syringeability at room temperature was measured by computing the work (mJ) required to expel the hydrogel loaded into a 1 mL syringe through a needle of 24G × 1.5″. Briefly, syringes were loaded with the optimized hydrogel, avoiding air bubble formation. Afterwards, syringes were placed vertically on a support and the punch of the texturometer descended at 2 mm/s, pressing the piston of the syringe a distance of 2 cm. All measurements were carried out six times.

Bioadhesion at 37 °C was analysed by placing 75 µL of each formulation on goat tanned leather, previously fixed in the lower support of the texturometer. Then, the texturometer punch, also covered with leather, descended at 1 mm/s and exerted a force of 0.5 N during 60 s over the sample. The area under the curve of the force–distance plot generated represented the bioadhesiveness (mJ) of the hydrogel. All measurements were carried out six times.

#### 2.3.2. Rheological Analysis

A rheometer AR1000-N (TA Instruments, Leatherhead, UK) was used to determine the gelation temperature (Tgel) and the hydrogel stability at 37 °C. The Tgel was calculated as the crossover of the storage (G′) and loss (G″) moduli of the formulation, using a conical geometry of 6 cm Ø and 2.1° at a fixed angular frequency of 5 rad/s and establishing a temperature ramp from 20 to 40 °C at 0.3 °C/min. To evaluate the stability of the hydrogel at body temperature, the G′ and G″ values were recorded at 37 °C at variable angular frequencies from 0.5 to 50 rad/s.

#### 2.3.3. Stability Assessments

The effect of storage on the texturometric and rheological parameters of the optimized hydrogel was re-evaluated at two weeks and one month at 4 °C.

### 2.4. Cell Cultures and hUCESC-CM Preparation

Culture of hUCESC was carried out as previously described [20]. Briefly, cells were cultured in 90 mm Petri dishes at 70% confluence in 5 mL of DMEM-F12 culture medium supplemented with FBS (10%) at 37 °C for 48 h in air-CO_2_ (95:5) atmosphere. Cells were washed three times in PBS and cultured again in 5 mL of DMEM-F12 without FBS. After 48 h, the medium was centrifuged for 5 min at 300*g*× to remove cellular debris, supernatant was collected, and used either immediately or lyophilized (SP Scientific, 25L Genesis 5 Q EL-85, Gardiner, MT, USA) and stored at −80 °C until used.

### 2.5. Hydrogel Loading with hUCESC-CM

Lyophilized hUCESC-CM (from 5 mL culture) and lyophilized DMEM-F12 medium (as control) were resuspended in 5 mL of the optimized hydrogel prepared in ddH_2_O at room temperature to obtain the hUCESC-CM-loaded hydrogel (H-hUCESC-CM) and the control hydrogel (H-DMEM-F12).

### 2.6. In Vivo Assay

#### 2.6.1. Ethics Statement

This study adhered to national regulations and was approved by the regional Ethics and Investigation Committee (Comité Ético de Investigación Clínica Regional del Principado de Asturias). Cervical smears were obtained from patients who underwent a routine gynecological check-up at Fundación Hospital de Jove, Asturias, Spain. All patients provided informed written consent. All animal studies were approved by the Universidade de Santiago de Compostela Ethics Committee for Animal Experiments (15010/17/006, 22 December 2017).

#### 2.6.2. Mice

Eight-week-old male C57BL/6 mice were purchased from the Central Animal Facility, Universidade de Santiago de Compostela (Santiago de Compostela, Spain). Mice were housed for three weeks before the experiment for adaptation and to acquire local bacterial flora before the experiment. Access to adequate water and food for mice was provided, and all the tests were carried out according to the approval of the Universidade de Santiago de Compostela Ethics Committee for Animal Experiments.

#### 2.6.3. Colitis Induction and Experimental Groups

**Figure 1:**
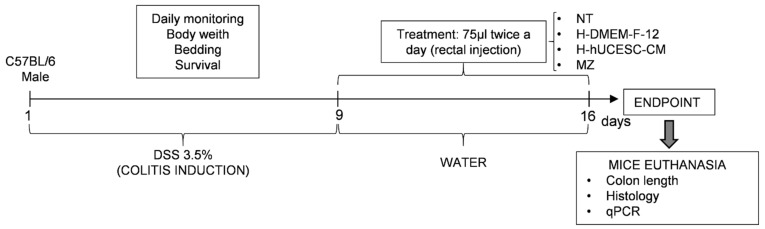
Study design for DSS induced colitis. Acute colitis was induced in C57BL/6 mice by administering 3.5% dextran sulfate sodium (DSS) in drinking water for nine days. Between 10 and 15 days, 75 μL of each treatment was rectally administered twice daily using a 26-gauge probe needle. On day 16 animals were sacrificed. NT: non treated, H-DMEM-F-12: DMEM-F12-loaded hydrogel; H-hUCESC-CM: H-hUCESC-CM-loaded hydrogel; MZ: mesalazine.

In vivo experiments were performed for 16 days according to Wirz et al. [30] (Figure 1). Acute colitis was induced in C57BL/6 mice by administration of 3.5% DSS in drinking water for nine days. Treatments were performed between days 10 and 15 by rectal administration of 75 μL twice a day. Before rectal administration, mice were anesthetized by inhalation of isoflurane (2.5% for induction and 1–2% for maintenance) using a calibrated vaporizer. Rectal administration was given with a 26-gauge probe needle with rounded tip inserted about 2 cm. Formulation was injected slowly, using the gel itself as a lubricating agent to impregnate the cannula so as not to cause damage in the intestinal wall and keeping the mouse immobile for a couple of minutes to ensure the immediate retention of the treatment.

Animals were divided into five groups of six mice per group, as follows: (a) healthy control group (H); (b) no-treatment group (NT): mice without treatment after DSS administration; (c) H-DMEM-F12 control group: mice treated with H-DMEM-F12 after DSS administration; (d) H-hUCESC-CM group: mice treated with H-hUCESC-CM after DSS administration; (e) mesalazine group (MZ): mice treated with mesalazine foam after DSS administration.

#### 2.6.4. Clinical Symptoms Evaluation and Colon Macroscopic Examination

Clinical signs of colitis, including survival, body weight, stool consistency, and bleeding severity were recorded. At the endpoint of the experiment, the intestine was removed, and the colon was separated from the small intestine. Colon length, including cecum, was measured.

#### 2.6.5. Histological Evaluation

Tissue sampling for histological analysis was performed by cutting three colonic fragments (0.5 cm each) with respect to cecum: proximal colon, mid colon, and distal colon. A piece (0.2–0.4 mm) from each fragment was fixed in 10% formalin and embedded in paraffin, and 5 μm thick paraffin tissue sections were cut, deparaffinized, rehydrated, and stained with hematoxylin and eosin (H&E) to be graded for intestinal epithelial changes by a blinded expert pathologist (LOG). Based on a previously published score [23], the following parameters were evaluated: degree of inflammation and cell infiltration (0, none; 1, slight; 2, moderate; 3, severe), microscopic ulcerations (0, none; 1, focal; 2, extensive), crypt architecture (0, normal; 3, severe crypt distortion with loss of entire crypts), epithelial atrophy (0, none; 1, slight; 2, moderate; 3, severe), and epithelial dysplasia (0, none; 1, low grade; 2, high). Histological damage score is the sum of each individual score. It should be noted that, unlike human UC, crypt abscesses are not characteristic of this model and are rarely seen.

#### 2.6.6. RNA Extraction and Quantitative Real Time PCR

Tumor necrosis factor-alpha (TNF-α), Interleukin 6 (IL-6), interferon-gamma (IFN-γ), and 18S (as control) mRNA expression levels were evaluated by real-time PCR. RNA extraction was performed using tissue from the most proximal zone. cDNA synthesis was carried out from 1.5 μg of RNA. Samples were denatured at 94 °C for 10 s, annealed at 58 °C for 10 s, and extended at 72 °C for 10 s for a total of 35 cycles. PCR products formed in each cycle were evaluated based on SYBR Green fluorescence with 18S as endogenous control. Primers sequences are shown in Table 1.

### 2.7. Statistical Analysis

In all experiments, mean ± standard error of the mean (SEM) was calculated. Experiments were performed at least in triplicate. One-way ANOVA followed by Tukey’s and LSD post-hoc tests were used for multiple comparison. PASW 18.0 and GraphPad Prism software were used for all calculations. Level of significance was *p* < 0.05.

## 3. Results

### 3.1. Hydrogel Preparation and Characterization

**Figure 2:**
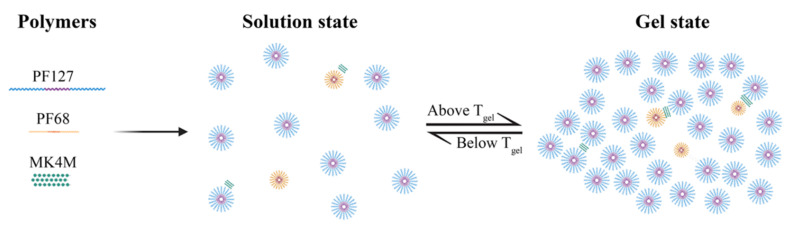
Potential structural formula of the formulated ternary hydrogel based on Pluronic^®^ F127, Pluronic^®^ F68, and hydroxypropyl methylcellulose.

Dispersions of Pluronic^®^ copolymers at low temperatures exist in the form of unimers even at high concentration. Upon heating, the dehydration of PPO blocks allows the association of the unimers to form micelles. The addition of PF68 to a dispersion of PF127 disturbs the formation of PF127 micelles. However, it has been described that mixtures of PF127 and PF68 do not lead to the formation of mixed micelles due to segregation of the copolymers [31,32]. HPMC can also interact with the PEO moiety of Pluronic^®^, reducing the number of polymer chains forming the micelles (Figure 2). Variations in the components proportions lead to the production of systems with tunable Tgel and properties.

Modelling of the experimental design results by ANN and the genetic algorithms allowed us to determine the composition of an optimized hydrogel using PBS as dissolution medium. The liquid texture of this hydrogel at room temperature facilitates application and extension on the rectal mucosa. In addition, its thermosensitive and bioadhesive properties favour gelation at body temperature and retention on the ulcerated epithelium.

Thus, the model predicted an optimized formulation composed of 16.34% of PF127, 2.55% of PF68, and 0.26% of MK4M in PBS. Both predicted and experimental data agree and are shown in Table 2. Despite the increase in values for syringeability, rectal administration is still possible using the 26-gauge probe needle.

**Figure 3:**
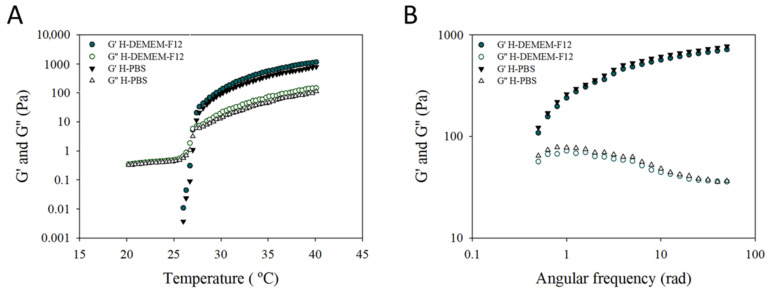
Variation of the storage (G′) and loss (G″) moduli as a function of temperature (**A**) and angular frequency at 37 °C (**B**) for H-DMEM-F12 and H-PBS. The crossover between both moduli is represented by Tgel.

As a step forward in pursuing H-hUCESC-CM delivery system, the optimized hydrogel formulation using the hUCESC culture media (DMEM-F12) was prepared to ensure adequate performance. Figure 3 shows the rheological properties of H-PBS and H-DMEM-F12. Temperature (A) and angular frequency (B) profiles for both hydrogels are superimposed, indicating their similitude. There are no statistically significant differences between H-DMEM-F12 and H-PBS characteristics. Table 3 shows the evolution of H-DMEM-F12 parameters during storage. No statistically significant differences were observed for syringeability work and Tgel over time. However, bioadhesion was significantly increased at two and four weeks of storage (*p* < 0.01).

### 3.2. Inducement of DSS-Associated Colitis in C57BL/6 Mice

In the DSS induced colitis model, mice showed sequentially initial, mild, and severe clinical signs of disease (weight loss, diarrhoea occurrence, and blood in stools) after three-day treatment with DSS. Macroscopic and microscopic lesions induced by DSS were confirmed in all mice. None of the mice died during the study and no side effects due to the H-hUCESC-CM administration were detected.

### 3.3. Effect of H-hUCESC-CM on Body Weight and Colon Length

**Figure 4:**
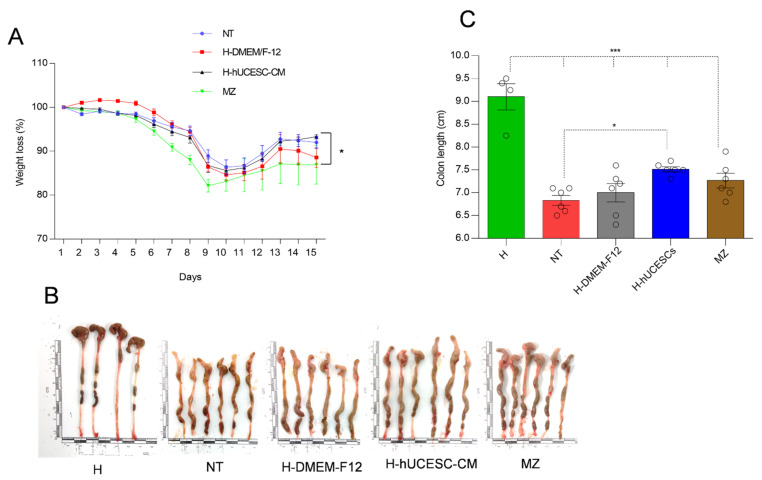
H-hUCESC-CM ameliorates DSS-induced colitis. (**A**) Daily body weight measurement as percentage of starting weight. Asterisk indicates a significant (*p* < 0.05) difference in body weight at day 15 between H-hUCESC-CM and MZ mice. (**B**) Colon length images at day 16. (**C**) Colon length values at day 16. DSS-induced colon shortening, however, treatment with H-hUCESC-CM significantly reduced these changes with respect to NT mice. Data are expressed as mean ± SEM. * = *p* < 0.05; *** = *p* < 0.001.

Weight measurements were performed daily. It is known that body weight loss is a key indicator of colitis development in DSS-treated mice [33]. As expected, DSS-treated mice lost weight rapidly, being the lowest on day nine, and then gradually recovering in all mouse groups (Figure 4A). The highest average weight recovery was in the H-hUCESC-CM group, which was significantly higher at 15 days than the MZ-treated group.

Administration of DSS causes a significant (*p* < 0.001) decrease in colon length in all groups of mice as compared to control H mice. Interestingly, only H-hUCESC-CM treatment significantly (*p* < 0.05) increased colon length with respect to the NT-group (Figure 4B,C).

### 3.4. H-hUCESC-CM Hydrogel Reduced the Extension and Severity of Intestinal Lesions

**Figure 5:**
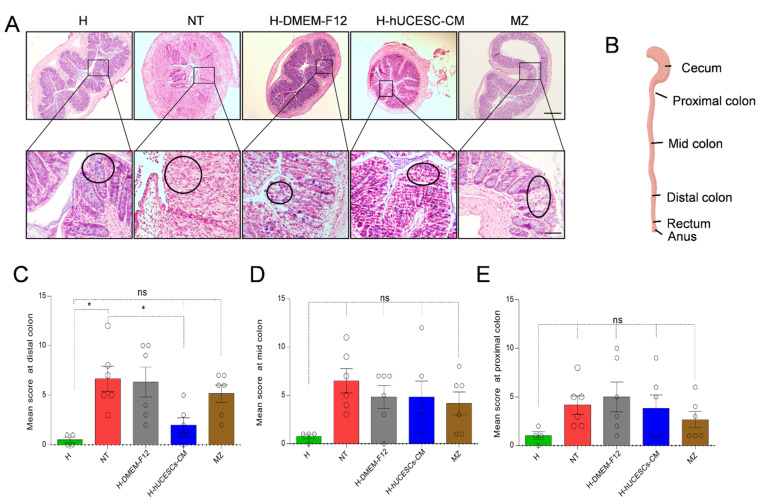
Histological evaluation of colon. (**A**) Representative mice colon sections stained with H&E. Microphotographs shown areas of normal epithelium (H), ulceration and severe inflammatory reaction (NT), glandular distortion (H-DMEM-F12), mild inflammation (H-hUCESC-CM), and atrophy (MZ). (**B**) Representative picture of histological analysis. Analyses were done in three colonic fragments: distal, mid, and proximal (with respect to cecum). (**C**) Colon epithelial score in the distal fragment (based on inflammation and cell infiltration, microscopic ulcerations, crypt architecture, epithelial atrophy, and epithelial dysplasia, see Methods). (**D**) Colon epithelial score in the middle fragment. (**E**) Colon epithelial score in the proximal fragment. Data are expressed as mean ± SEM. H: healthy mice, NT: non treated mice, H-DMEM-F-12: DMEM-F12-loaded hydrogel treated mice; H-hUCESC-CM: H-hUCESC-CM-loaded hydrogel treated mice; MZ: mesalazine-treated mice. * = *p* < 0.05; ns = not significant. Scale bar: 500 μm for low (upper) and 100 μm for high (below) magnification.

Colon evaluation by H&E showed that DSS treatment in NT mice induced a high degradation of mucosal architecture, inflammatory cell infiltration, ulceration, edema, destruction of epithelial cells, and diffused depletion of goblet cells, as well as intense collapse of crypts. Figure 5A shows representative examples of histopathology analysis performed after H&E staining. These changes were significantly reduced in the H-hUCESC-CM treated mice, which presented only limited colonic mucosal congestion and edema, scattered erosions, healing ulcers, and less inflammatory cell infiltration. Mice from the H group showed normal mucosal architecture and regular goblet cells (Figure 5A).

Histological score values in three colon locations, with respect to cecum (proximal, mid, and distal) (Figure 5B), indicate that H-hUCESC-CM administration reduces histological score in all locations with respect to NT mice; however, differences were only statistically significant for distal location (Figure 5C–E). As expected, the highest histological score in this location was for the NT group, which was significant (*p* < 0.05) with respect to the H mice. Mice treated with H-hUCESC-CM also showed significantly (*p* < 0.05) reduced scores with respect to NT mice. Though not significant, reduced scores were observed in H-hUCESC-CM-treated mice with respect to the NT group in all the other colon locations.

**Figure 6:**
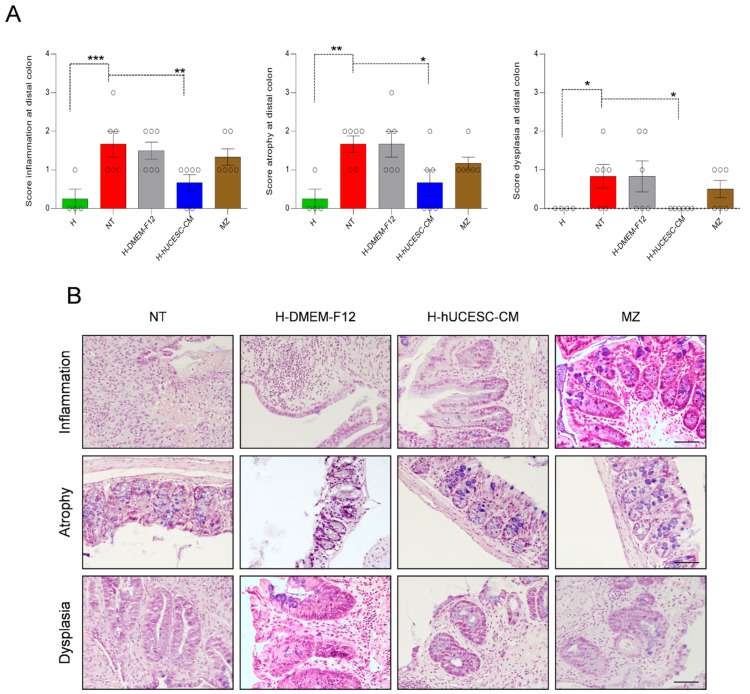
Representative histological score in the distal colon fragment. (**A**) Score values of inflammation, atrophy, and epithelial dysplasia in healthy (H), and treated and non-treated DSS-induced mice. (**B**) Microphotography of representative examples of inflammation, atrophy, and dysplasia in DSS-induced mice, non-treated (NT) and treated with H-DMEM-F12, H-hUCESC-CM, and mesalazine (MZ). * = *p* < 0.05; ** = *p* < 0.01; *** = *p* < 0.001. Scale bar: 100 μm.

Statistical differences within groups were also found with respect to specific histopathological parameters, such as inflammation, atrophy, and epithelial dysplasia. Specific scores for these parameters in the distal colon were significantly lower with respect to NT mice (*p* < 0.01, *p* < 0.05, and *p* < 0.05, respectively) after treatment with H-hUCESC-CM (Figure 6A). An example of inflammation, atrophy, and dysplasia in NT, H-DMEM-F12, H-hUCESC-CM, and MZ mouse groups after H&E staining is shown in Figure 6B.

### 3.5. H-hUCESC-CM Proinflammatory Cytokines in Colon

**Figure 7:**
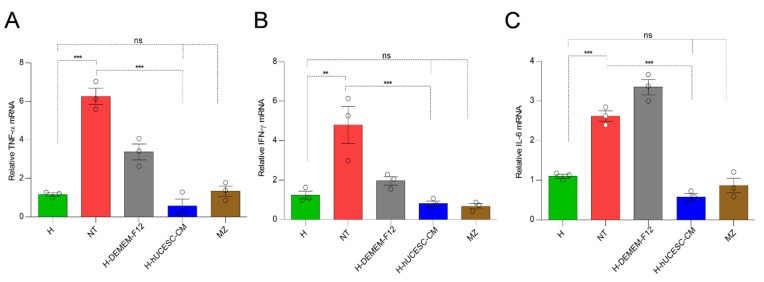
Real-time PCR of pro-inflammatory cytokines in colon samples. (**A**) Tumor necrosis factor-alpha (TNF-α), (**B**) Interferon-gamma (IFN-γ), and (**C**) Interleukin-6 (IL-6) mRNA expression levels in colon of mice. Data are expressed as mean ± SEM. ns = not significant. ** = *p* < 0.01; *** = *p* < 0.001.

TNF-α, IL-6, and IFN-γ mRNA expression levels in the different mouse groups are shown in Figure 7. TNF-α, IFN-γ, and IL-6 mRNA expression levels were significantly higher in the NT mice with respect to H mice (*p* < 0.0001, *p* < 0.01, and *p* < 0.0001, respectively). All the cytokines evaluated significantly decreased after treatment with H-hUCESC-CM as compared with NT mice (*p* < 0.0001). Importantly, TNF-α, IFN-γ, and IL-6 mRNA expression levels in H mice were similar (not significantly) to both H-hUCESC-CM and MZ treated mice.

## 4. Discussion

Study results demonstrate that local administration of H-hUCESC-CM has therapeutic effects in an acute DSS-induced colitis mouse model. These beneficial effects comprise body weight recovery, improved colon length, improved histological colitis score, and decreased pro-inflammatory cytokine expression.

Mucosal membranes are excellent sites for drug delivery owing to their high permeability, lower risk of overdose, and local treatment. However, the major drawback of drug delivery via mucosal membranes is the limited retention time at the mucosal tissue surface [34,35].

The use of ANN made it possible to obtain an optimized bioadhesive hydrogel that was stable and suitable for rectal administration of hUCESC-CM. Its mucoadhesive and thermosensitive character, together with its suitable syringeability, ensures adequate administration and extensibility in liquid form on the colonic tissue. The low Tgel (27 °C) of H-DMEM-F12 enables a faster transition from liquid to solid state by the Pluronic^®^ polymeric chains rearrangement, reducing leakage and dilution of the systems in biological fluids [36]. Moreover, the incorporation of MK4M improves hydrogel bioadhesion for a longer and closer interaction between hUCESC-CM and colonic and rectal mucosae [28,37]. Thus, the residence time of the drug delivery system in the colon increases, enhancing drug absorption, as well as efficacy and bioavailability.

With respect to NT mice, it was found that H-hUCESC-CM treatment significantly improved several histopathological parameters, such as colon length and histological score. Specifically, the lower grade of atrophy, inflammation, and dysplasia suggests that H-hUCESC-CM acts upon several targets. It is known that molecular and cellular mechanisms responsible for MSC-mediated attenuation of murine colitis involved suppression of colon inflammation, promotion of angiogenesis, and regeneration of damaged epithelium, leading to an enhanced healing process in the injured colon [38,39,40]. It is worth noting that, compared to the standard formulation (MZ), treatment with H-hUCESC-CM improved histological score only at the distal colon, which is interesting because histopathological damage by DSS is predominantly found in the distal colonic location [41]

It has been previously demonstrated in a dry eye rat model that hUCESC-CM treatment significantly improves epithelial regeneration and decreases pro-inflammatory cytokines [21]. hUCESC-CM contains high levels of proteins, such as tissue inhibitors of metalloproteinases-1 and -2, fibroblast growth factor -6 and -7, urokinase receptor, and hepatocyte growth factor, that could mediate regenerative effects [20,21,22,23]. In the present study, mice treated with H-hUCESC-CM showed a significant decrease in TNF-α, IL-6, and IFN-γ mRNA expression. TNF-α is considered the most potent pro-inflammatory cytokine in the pathogenesis of IBD. In fact, anti-TNF-α agents, such as infliximab, are highly effective in the treatment of moderate to severe intestinal disease [42]. Experimental data show that hydrogel alone also conferred a partial benefit on some pro-inflammatory cytokines, such as TNF-α and IFN-γ; however, this may be due to properties of the polymers included in the formulation [43,44].

Earlier studies attributed the effects of MSC therapies to their capacity for local engrafting and differentiating into multiple tissue types; however, more recent studies show that implanted cells do not survive for long [45,46]. This suggests that the beneficial effects of MSC therapy could be derived from secreted bioactive factors regulating key biologic processes [47]. Therefore, secretome derivatives, such as CM or exosomes, may be preferable to cell administration in terms of manufacturing, storage, and handling [48]. The use of MSC-CM mitigates certain safety considerations related to the transplantation of living cells such as immune compatibility, emboli formation, tumorigenicity, and transmission of infections [48]. One limitation of this study was that only a single concentration of H-hUCESC-CM was evaluated. It is known that the effectiveness of this type of therapy may vary depending on the dose and frequency of administration. Further research is necessary to elucidate these aspects, as well as the underlying mechanisms and components of hUCESC-CM.

## 5. Conclusions

The present study demonstrates that local administration of hUCESC-CM in the form of bioadhesive thermosensitive hydrogels improves residence time in target sites and increases interaction with rectal and colonic mucosae, inducing therapeutic anti-inflammatory and regenerative activity in experimental colitis. The data suggest that H-hUCESC-CM may represent an attractive tool for cell-free therapy in IBD local treatment.

## 6. Patents

Title: “Human uterine cervical stem cell population and uses thereof”. Inventors; Francisco J. Vizoso, Roman Pérez-Fernandez and Noemi Eiró. Holder: GiStem Research S.L. EP 2 770 050 B1, filed 22. 02. 2013, granted 16. 11. 2016. WO/2014/128291.

## Figures and Tables

**Table 1:** Primer sequences designed to evaluate TNF-a, IFN-g, IL-6, and 18S (as control) mRNA by RT-PCR.

Gene	Forward	Reverse
TNF-α	aggctgccccgactacgt	gactttctcctggtatgagatagcaaa
IFN-γ	cagcaacagcaaggcgaaa	ctggacctgtgggttgttgac
IL-6	acaagtcggaggcttaattacacat	ttgccattgcacaactctttt
18S	cccctcgatgactttagctgagtgt	cgccggtccaagaatttcacctct

**Table 2:** Texturometric and rheological values predicted by the ANN model and experimental values obtained after hydrogel characterization. Data are presented as mean ± SEM.

Parameter	Predicted by ANN	H-PBS
Syringeability work (mJ)	141.92	170 ± 0.95
Bioadhesion work (mJ)	0.33	0.31 ± 0.02
T_gel_ (°C)	27.2	26.9 ± 0.20

**Table 3:** Properties of the H-DEMEM-F12 after variable storage time at 4 °C. Data are presented as mean ± SEM.

Parameter	Day 0	2 Weeks	4 Weeks
Syringeability work (mJ)	176.17 ± 1.30	182.15 ± 1.64	175.79 ± 1.19
Bioadhesion work (mJ)	0.270 ± 0.01	0.327 ± 0.03	0.520 ± 0.03
T_gel_ (°C)	26.89 ± 0.07	26.77 ± 0.18	26.17 ± 0.20

## Data Availability

Not applicable.
